# Should surgery be conducted for small nonfunctioning pancreatic neuroendocrine tumors: a systematic review

**DOI:** 10.18632/oncotarget.15685

**Published:** 2017-02-25

**Authors:** Jingfei Guo, Jianjun Zhao, Xinyu Bi, Zhiyu Li, Zhen Huang, Yefan Zhang, Jianqiang Cai, Hong Zhao

**Affiliations:** ^1^ Chinese Academy of Medical Sciences, Peking Union Medical College, Beijing, China; ^2^ Department of Abdominal Surgical Oncology, Cancer Hospital, Chinese Academy of Medical Sciences and Peking Union Medical College, Beijing, China

**Keywords:** pancreatic neuroendocrine tumor, 2 cm, surgical resection

## Abstract

**Background:**

The incidence of nonfunctioning pancreatic neuroendocrine tumors smaller than 2cm has increased remarkably in the last two decades. Controversies exist regarding whether surgery should be conducted for this group of tumors.

**Methods:**

MEDLINE, EMBASE and CENTRAL were search until 2017/01/17. Studies with comparative results between operation and observation group were included. Primary outcomes were overall survival and disease specific survival. Secondary outcomes were disease progression and surgical death and complications.

**Results:**

6 studies with a total of 1861 patients were identified. No randomized controlled trials were found. Survival rate was high (97-100%) and no patients died because of the disease in 5 of the 6 studies, with no difference between operation and observation group. Disease progression was compared in 3 of the 6 studies. 2 studies reported minimal disease progression (0-3.5%) and no significant difference between operation and observation group. Perioperative deaths were rare (0-3%), but complications were common (33-46%). None of the 46 patients who crossed over form observation to operation group had disease recurrence after resection.

**Conclusion:**

Small NF-PNETs without distant metastasis, lymph node metastasis and local invasion on imaging studies can be observed without increase in death and disease progression.

## INTRODUCTION

Nonfunctioning neuroendocrine tumors of the pancreas (NF-PNETs) refers to PNETs without clinical symptoms of hormonal hypersecretion [1]. Although considered rare, the patient population is constantly growing. From 1998 to 2011, small (size < 2cm) NF-PNETs increased three folds as a proportion of all PNETs, making up to 20% of all PNETs cases in 2011 [2]. This is probably due to more gastroenterology investigations for preventive purpose and the use of high-resolution imaging [3–6]. Most small NF-PNETs are indolent [7]. But a small proportion of them are malignant, even those smaller than 0.5cm [2, 8].

This hard-to-predict behavior cause controversy in its management. Both the ENETS and NCCN guideline recommend routine surgical resection for localized NF-PNETs larger than 2cm, given the significant survival benefit [9]. As for NF-PNETs smaller than 2cm, no definite conclusion has been reached. The latest ENETS guideline quoted “a non-operative approach could be advocated in selected cases for tumors≤2 cm that are discovered incidentally” [1]. The NCCN 2015 guideline quoted “includes observation alone as an option for selected cases of incidentally discovered neuroendocrine tumors measuring 1cm, but recommends surgical resection for larger tumors absent indications” [10].

In this study, we aim to collect all the existing evidence concerning whether small NF-PNETs need operation. To our knowledge, this is the first systemic review on the topic.

## RESULTS

### Basic characteristics of studies and quality assessment

**Figure 1:**
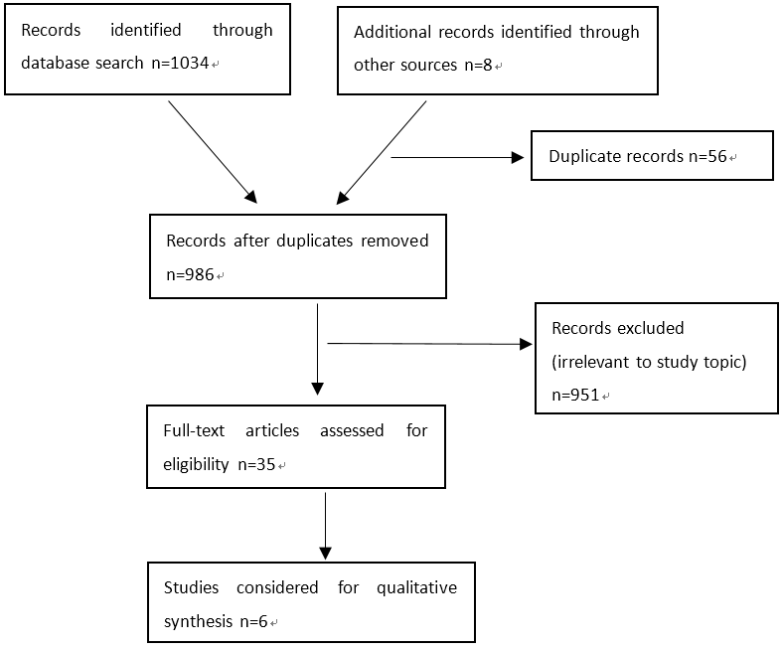
PRISMA diagram showing article selection for the review

As shown in Figure 1, our literature search identified 986 unique references. Detailed search strategy can be found in Supplementary Table S1 online. After full text review of 35 manuscripts, we found six studies [11–16] with a total of 1861 patients in accordance with the inclusion criteria. No randomized controlled trials were identified. Five of the six researches were retrospective cohort studies and one was a case-control study. Quality of the six studies was assessed by NOS, as shown in Supplementary Table S2 online. Five of them were high quality research scored ≥ 6. One study (Gratian) scored 5, indicating medium quality.

From Table 1, we can see that not all studies strictly met with patient exclusion criteria. All studies included patients with NF-PNETs smaller than 2cm and excluded familial syndromes associated with PNETs. But only two of them (Sadot and Jung) ruled out all signs of metastasis and invasion before operation. One study (Lee) didn't exclude lymph node metastasis. One study (Rosenberg) excluded neither lymph node metastasis nor local invasion, but ruled out G3 tumors instead. Two studies (Gratian and Regenet) didn't exclude distant metastasis, lymph node metastasis or local invasion. Because of this heterogeneity, we conducted qualitative other than quantitative synthesis of survival information across studies.

**Table 1:** Basic characteristics of studies included

Author	Study design	Study duration	Study location	Study size	Median follow up (months)	Distinct exclusion criteria*	Primary outcome	Secondary outcome
Lee, 2012 [11]	Retrospective cohort study	2000-2011	Unites States	101	Operation group: 52 Observation group: 45	Distant metastasis; Local invasion	Disease specific survival	Surgical death and complications
Gratian, 2014 [12]	Retrospective cohort study	1998-2011	Unites States	1367	62.4	/	5-year OS	/
Jung, 2015 [13]	Retrospective cohort study	1995-2012	North Korea	145	G1 tumor: 32.9 G2/3 tumor: 39.3	Distant metastasis; Local invasion; Regional lymphnode metastasis	Overall survival rate	Disease progression
Regenet, 2016 [14]	Retrospective cohort study	1999-2012	France	80	34.0	/	Overall survival rate	Disease progression; Surgical death and complications
Sadot, 2016 [15]	Case control study	1993-2013	United States	150	Operation group: 57 Observation group: 44	Distant metastasis; Local invasion; Regional lymphnode metastasis	Disease specific survival	Surgical death and complications
Rosenberg, 2016 [16]	Retrospective cohort study	1999-2014	United States	18	27.8	Distant metastasis; G3 tumor	Overall survival rate	Disease progression; Surgical death and complications

* All 6 studies included sporadic PNETs only and excluded familial syndromes, so we only listed other exclusion criteria in the table.

### Primary outcomes

We can see from Table 2 that four studies (Gratian, Jung, Regenet, Rosenberg) provided data on overall survival and two studies (Lee, Sadot) on disease specific survival. Except for the one conduct by Gratian et al., all other studies had remarkably good survival results. Either overall survival rate or disease specific survival was nearly 100%, and no significant difference existed between operation and observation group. In Gratian's research, 5-year overall survival was 72.3%-86% in the operation group and merely 27.6% in the observation group. The survival difference was statistically significant on univariate analysis, and no multivariate analysis was conducted.

**Table 2:** Primary and secondary outcomes for all patients

Author	Patients number		Survival			Disease progression			Surgical death and complications	
	OG	NOG	OG	NOG	Statistical significance	OG	NOG	Statistical significance	Death	Complications
Lee [11]	26 (25.7%)	75 (74.3%)	Disease specific survival: 100%	Disease specific survival: 100%	not significant	/	/	/	0%	Overall: 46% POPF*: 34% Grade B/C: 27%
Gratian [12]	999 (73%)	368 (27%)	5-year OS 72.3%-86.0%**	5-year OS: 27.6%	P<0.01 univariate analysis	/	/	/	/	/
Jung [13]	60 (41.4%)	85 (58.6%)	Overall survival rate: 100.0%	Overall survival rate: 100.0%	not significant	0%	3.5%	not significant	/	/
Regenet [14]	66 (82.5%)	14 (17.5%)	Overall survival rate: 97.0%	Overall survival rate: 100.0%	not significant	11%	14%	not significant	3%	Overall: 44% POPF*: 29% Grade B/C:20%
Sadot [15]	60 (40%)	90 (60%)	Disease specific survival: 100%	Disease specific survival: 100%	not significant	/	/	/	0%	Overall: 33% POPF*: 21%
Rosenberg [16]	8 (44.4%)	10 (55.6%)	Overall survival rate: 100.0%	Overall survival rate: 100.0%	not significant	0%	0%	not significant	0%	Overall: 35% POPF*: 25%

* POPF=postoperative pancreatic fistulas

** 72.3% for pancreaticoduodenectomy, 83% for partial pancreatectomy, 86% for total pancreatectomy

### Secondary outcomes

As shown in Table 2, three studies (Jung, Regenet, Rosenberg) reported data on disease progression. No significant difference existed between operation and observation group in all three studies. In Rosenberg's research, no patients had disease progression. In Jung's study, no patients had disease recurrence after surgery and 3 out of 85 patients (3.5%) had disease progression during observation. All three patients had meaningful tumor growth (≥20% or ≥5mm) without metastasis. In Regenet's study, 11% and 14% of patients had disease progression in operation and observation group respectively. In operation group, 7 patients (11%) developed metastasis after resection. In observation group, 2 patients (14%) developed metastasis during follow-up without increase in size of the primary tumor.

Four studies (Lee, Regenet, Sadot, Rosenberg) reported surgical death and complications (Table 2). Very few patients died within the perioperative period but postoperative complications were quite common. The only two death cases were from Regenet's study. Both patients had pancreaticoduodenectomy. Postoperative complications happened in 33-46% patients, with postoperative pancreatic fistulas (POPF) being most common (21-34%).

### Outcomes for crossover patients

In Table 3, we summarized information on patients who were originally included in the observation group and later crossed over to the operation group.

**Table 3:** Outcomes for crossover patients and follow-up information

Author	Follow-up interval and methods	Follow-up period before crossover	Number to crossover (% in NOG)	Reason to crossover (patient number) or not to crossover	Survival and disease progression of the crossover group
Lee [11]	CT/MRI imaging at 3/6-month interval	60 months 36 months	2 (2.6%)	Develop pancreatic duct dilatation (1) Patient's/physician's preference (1)	Overall survival: 100% Disease recurrence: 0%
Gratian [12]	/	/	/	/	/
Jung [13]	Imaging studies at 3/6/12-month interval (depending on tumor morphology and size)	/	12 (14.1%)	Increase in tumor size (8) Meaningful tumor growth(2) Develop symptom (1) Patient's preference (3)	Disease specific survival 100% Disease recurrence: 0%
Regenet [14]	/	/	0	2 patients had disease progression in NOG, both developed metastasis	/
Sadot [15]	Clinic visits interval≤3months ≥2 imaging studies	30 months (7-135 months)	26 (25.0%)	Increase in tumor size (8) Develop pancreatic duct dilatation (1) Patient's/physician's preference (17)	Overall survival: 92.3% Disease specific survival 100% Disease recurrence: 0%
Rosenberg [16]	CT/MRI imaging start at 6-month interval	/	0	No disease progression in NOG	/

Three of the six studies had crossover patients during follow-up. None of them had disease recurrence after operation or died of the disease. In Lee's study, 2 patients (2.6%) transferred to the operation group. One had surgery for development of pancreatic duct dilatation, and the other because of physician's preference. In Jung's study, 12 patients (14.1%) crossed over to resection group. 8 of them had increase in tumor size, one developed symptoms, and the other 3 had surgery due to their own preference. In Sadot's study, 26 patients (25%) transferred to the operation group. 8 of them had increase in tumor size, one had development of pancreatic duct dilatation, and 17 patients had surgery due to their own will or physician's preference.

In Rosenberg's study, no patients transferred to the operation group. No signs of disease progression occurred, and both patients and physicians were willing to stick to the wait-and-see policy. In Regenet's study, 2 patients in the observation group had disease progression, both presented in the form of metastasis without growth of the primary tumor. Neither had surgery because of the metastatic lesions. In Gratian's research, 1001 out of 1367 patients were lost at the beginning of the follow-up and survival analysis was conducted for 366 patients only. For these 366 patients, no information concerning follow-up or crossover was reported.

## DISCUSSION

Two decades ago, small NF-PNETs were rarely found. Since then, the incidence has gone through a sharp increase and controversies arise in surgical management. Some physicians emphasized on the malignant potential and recommended resection upon tumor detection. They collected information from patients who had surgery and found that some small NF-PNETs had malignant pathology and developed metastasis [8, 17–20]. Other physicians believed that these tumors can be safely observed for a while and operation did more harm than good. They adopted wait-and-see policy for selected patients and got good survival results [7]. But single-arm studies were inevitably biased. In the last five years, more rigorous studies presenting comparative outcomes for both operation and observation group were conducted. In this paper, we summarized results from all comparative studies and tried to draw a conclusion.

Survival rate was high (97-100%) and no patients died because of the disease in 5 of the 6 studies, with no difference existed between the operation and observation group. The only exception was Gratian's research. The operation group had a 5-year overall survival of 72.3-86%, while the observation had a merely 27.6%. Disease progression was compared in 3 of the 6 studies. Two studies reported minimal disease progression (0-3.5%) and no significant difference between the operation and observation group. Regenet's research, however, reported much higher disease progression rate up to 11.2%, though the difference was also not significant between operation and observation. Gratian's and Regenet's studies were divergent from the others which demonstrated that small NF-PNETs had equally good survival and minimal disease progression under both operation and observation.

This divergence can be explained from two aspects. First is patient characteristics. We can see from Table 1 that the other four studies all ruled out high-risk patients. All of them excluded patients with distant metastasis. Three of them also excluded patients with lymph node metastasis or local invasion, and the other excluded G3 patients if pathology results were available. Neither Gratian's nor Regenet's research set such exclusion criteria. In Regenet's study, 4 patients (5%) had synchronous metastasis at the beginning of follow-up, and no information on lymph node status or local invasion was provided. This could explain the much higher disease progression rate. In Gratian's study, 173 patients (12.6%) had distant metastasis, 472 patients (34.5%) had lymph node metastasis, and no data on local invasion was reported. Also, patients in the observation group had more lymph node metastasis, larger tumor size, older age, worse hospital care and less chemotherapy compared to the operation group. With all those significant differences uncontrolled, it was invalid to conclude that operation led to better overall survival than observation.

The second factor is follow-up. We can see from Table 3 that 5 of the 6 studies provided detailed follow-up information, except Gratian's study. Whether patients were closely followed up and how many patients in the observation group crossed over to the operation group were not reported. It also failed to report whether patients remained in the observation group due to no disease progression or unresectable lesion. The conclusion that surgical resection was associated with improved survival could not be drawn without adequate follow-up information.

Four studies reported surgical death and complications, and outcomes were unanimous. Perioperative deaths were rare but postoperative complications were common. The most common type of complication was POPF. According to ISGPF [21], Grade B POPF which may cause readmission and Grade C POPF which may cause death were not unusual*(20-27%)*. The result was consistent with previous findings. Smith et al. analyzed 2274 PNETs patients who had surgery from 1998 to 2006. They found that mortality rate was 1.7% and overall complications were present in 29.6% of patients after pancreaticoduodenectomy, total pancreatectomy and partial pancreatectomy as a whole [22]. Gooiker et al studied PNETs patients who underwent pancreaticoduodenectomy from 2000 to 2009, and reported that 90-day mortality rate varied from 4.8% to 7.4% [23]. Atypical resection such as enucleation caused less mortality, but overall complication and POPF rate were still high. According to Brient et al, 36.5% of patients suffered postoperative complications and 27% had POPF after enucleation [24].

As several studies have proved, a small proportion of small NF-PNETs are malignant. For these tumors, will delayed operation harm survival? The answer for this question can be found in patients who crossed over from observation group to operation group during follow-up. From Table 3, we can identity 40 crossover patients from 3 studies. 21 of them had operation due to patient's/physician's preference without disease progression. 19 of them had operation because of increase in tumor size, development of symptoms or pancreatic duct dilatation. Among them, 2 patients had meaningful tumor growth (≥20% or ≥5mm). None of the 40 patients died because of the disease and none of them had disease recurrence after operation. These data suggested that delayed surgical intervention may not compromise survival, even for those who developed disease progression during follow-up.

From the above analysis, we can conclude that for small NF-PNETs, selected patients can be observed without increase in death and disease progression, and that operation caused unnecessary death and complications. To select suitable patients for observation, we need to exclude distant metastasis, lymph node metastasis and any sign of local invasion on imaging studies. For patients who have pathological diagnosis, G3 tumors are malignant and should be excluded from observation. For patients who did not have pathological diagnosis, fine needle aspiration (FNA) is not obligatory, as long as the above results were concerned. FNA is a useful method for diagnosis of PNETs with excellent sensitivity and specificity [25–27], but its accuracy of determining histological grade is impaired. According to World Health Organization 2010 recommendations [28], analysis of 2000 cells is required for Ki-67 determination and at least 40 high-power fields for mitotic count, which is usually not possible for most FNAs. More evidences are needed to evaluate the necessity of FNA before adopting an observation approach.

Close follow-up is the key to identify tumors with malignant potential. Any sign of growth suggests more aggressive tumor type and should prompt the necessity of surgery. None of the six studies above had adequate regulation on follow-up interval, follow-up period and type of imaging tests required. Imaging studies at 3/6/12-month interval was required for all studies. In Rosenberg's study, follow-up interval started as 6 months. If elevation in tumor markers occurred, interval was decreased from 6 to 3 months. If no significant change happened, follow-up was continued for another 6 months, and then interval was increased to 1 year. We consider this follow-up interval to be reasonable. CT and MRI were the most commonly used imaging tests. The sensitivity to detect PNETs is approximately the same for CT and MRI, using comparable technical standards and equipment [29]. The ENETs guideline quoted“The decision whether to use CT or MRI depends on the preference, skill and expertise of the radiologist and the availability of the different techniques at each institution.” Given the frequency of imaging tests, we favor MRI without radiation exposure. Little data on follow-up period was provided. In Regenet's study, 26 patients crossed over to surgery after a median follow-up of 30 months, and we consider a minimum follow-up of three years is required. More researches are needed to specify follow-up strategy.

After excluding distant metastasis, lymph node metastasis and local invasion on imaging studies, small NF-PNETs can adopt a wait-and-see policy without increase in death and disease progression. More evidences are needed to specify follow-up strategy and whether FNA is required for decision making.

## MATERIALS AND METHODS

### Inclusion criteria

Two reviewers independently screened the studies according to specific inclusion and exclusion criteria. Inclusion and exclusion of contentious studies were made in consultation with a third reviewer.

#### Patients

Patients with NF-PNETS smaller than 2cm were considered for analysis. Diagnosis was made either according to imaging test or pathological results when surgery or fine needle aspiration (FNA) was conducted. Exclusion criteria included: 1) preoperative radiographic signs of local invasion, lymph node or distant metastasis 2) familial syndromes associated with PNETS, including multiple endocrine neoplasia or Von Hippel Lindau.

#### Intervention

All surgical procedures including total pancreatectomy, partial pancreatectomy, pancreaticoduodenectomy, distal pancreatectomy (with splenectomy) and enucleation were included.

#### Comparison

Two distinct groups, operative group (OG) and non-operative group (NOG), as well as explicit comparison of outcome between the two groups were required.

#### Outcome

Primary outcomes were overall survival (OS)and disease specific survival (DSS). Secondary outcome was disease progression defined by RECIST 1.1, as well as surgical death and complications.

#### Types of studies

Studies were included regardless of language, publication status or sample size. We intended to analyze randomized controlled trials (RCTs), quasi-RCTS and non-RCTs, but given the likely paucity of high-quality researches on the topic, cohort studies and case-control studies were also considered for analysis. Case series, case reports and other observational studies were excluded.

### Search strategy

Medline, EMBASE and CENTRAL were searched for relevant studies according to the above criteria until 2017/01/17. The computer search was supplemented with a manual search of primary studies referenced in all the retrieved articles. Oral reports from meetings and correspondence were also explored to minimize publication bias. If certain cohort was used in more than one studies, only the most recent and complete version was included. Full search strategies are displayed in Supplementary Table S1 online. The methodology was developed from the Preferred Reporting Items for Systematic Reviews and Meta-Analyses (PRISMA) statement.

### Data extraction

Two authors independently extracted relevant data including study design, study duration, study size, median follow-up time and year of publication, sex, age, tumor size, histology, biochemical markers, OS, DSS, PFS, disease recurrence/progression.

### Quality assessment

Study quality was assessed by JADAD score for RCTs and NOS for cohort studies/case-control studies. In the event of disagreements, consensus was achieved in discussion with the corresponding author.

### Statistical analysis

Meta-analysis was planned using Revman 5.3 with the following methods: calculation of the relative risk with 95 per cent confidence interval for dichotomous variables, calculation of the mean difference for continuous variables, use of a random effects model, evaluation of heterogeneity by χ^2^ test, and measure of the quantity of heterogeneity by means of the I^2^ value.

## SUPPLEMENTARY MATERIALS FIGURES AND TABLES


