# Supplementary material for: Should surgery be conducted for small nonfunctioning pancreatic neuroendocrine tumors: a systematic review
Source: Oncotarget. 2017 Feb 25;8(21):35368–75. doi: 10.18632/oncotarget.15685 (PMC5471061; doi:10.18632/oncotarget.15685)
Supplement: Supplementary file 1 [file oncotarget-08-35368-s001.pdf]

Should surgery be conducted for small nonfunctioning pancreatic neuroendocrine tumors: a systematic review

Supplementary Material

Supplementary Table S1 Database Search Strategy

Searched until 2017/01/17

Medline Search Results		
1#	neuroendocrine tumor OR neuroendocrine tumors OR neuroendocrine tumour OR neuroendocrine tumours OR adenoma OR adenomas OR apudoma OR Apudomas OR Carcinoma, Islet Cell OR Adenoma, Islet Cell OR beta-Cell Tumor OR Tumors, Island Cell OR Islet Cell Tumors OR Tumor, Island Cell OR Tumor, Islet Cell OR Islet Cell Adenoma OR Islet Cell Carcinoma OR Pancreatic Endocrine Tumour OR Islet Cell Tumor, Ulcerogenic	342661
2#	2cm OR 2 cm OR 2.0cm OR 2.0 cm	220331
3#	Operative Surgical Procedure OR Operative Surgical Procedures OR Procedures, Operative Surgical OR Surgical Procedure, Operative OR Operative Procedures OR Operative Procedure OR Procedure, Operative OR Procedures, Operative OR Procedure, Operative Surgical OR Pancreatectomy OR Pancreatectomies OR Pancreaticoduodenectomy OR Pancreatoduodenectomies OR Duodenopancreatectomy OR Duodenopancreatectomies OR Enucleation	4013917
4#	(((((((((randomized controlled trial [Publication Type]) OR controlled clinical trial[Publication Type]) OR randomized[Title/Abstract]) OR placebo[Title/Abstract]) OR randomly[Title/Abstract]) OR trial[Title/Abstract]) OR groups[Title/Abstract]) OR drug therapy[MeSH Subheading]) AND human[MeSH Terms])) OR cohort	3311925
5#	1# AND 2# AND 3# AND 4#	785
EMBASE Search Results		
1#	“neuroendocrine tumor” OR “neuroendocrine tumors” OR “neuroendocrine tumour” OR “neuroendocrine tumours” OR “adenoma” OR “adenomas” OR “apudoma” OR “apudomas” OR “Carcinoma, Islet Cell” OR “Adenoma, Islet Cell” OR “beta-Cell Tumor” OR “Tumors, Island Cell” OR “Islet Cell Tumors” OR “Tumor, Island Cell” OR “Tumor, Islet Cell” OR “Islet Cell Adenoma” OR “Islet Cell Carcinoma” OR “Pancreatic Endocrine Tumour” OR “Islet Cell Tumor, Ulcerogenic”	169,762
2#	“2cm” OR “2 cm” OR “2.0cm” OR “2.0 cm”	51,370
3#	“Operative Surgical Procedure” OR “Operative Surgical Procedures” OR “Procedures, Operative Surgical” OR “Surgical Procedure, Operative” OR “Operative Procedures” OR “Operative Procedure” OR “Procedure, Operative” OR “Procedures, Operative” OR “Procedure, Operative Surgical” OR Pancreatectomy OR Pancreatectomies OR Pancreaticoduodenectomy OR Pancreatoduodenectomies OR Duodenopancreatectomy OR Duodenopancreatectomies OR Enucleation	57,510
4#	1# AND 2# AND 3#	201
CENTRAL Search Results		
1#	neuroendocrine tumor OR neuroendocrine tumors OR neuroendocrine tumour OR neuroendocrine tumours OR adenoma OR adenomas OR apudoma OR Apudomas OR Carcinoma, Islet Cell OR Adenoma, Islet Cell OR beta-Cell Tumor OR Tumors, Island Cell OR Islet Cell Tumors OR Tumor, Island Cell OR Tumor, Islet Cell OR Islet Cell Adenoma OR Islet Cell Carcinoma OR Pancreatic Endocrine Tumour OR Islet Cell Tumor, Ulcerogenic	2664
2#	2cm OR 2 cm OR 2.0cm OR 2.0 cm	40332
3#	Operative Surgical Procedure OR Operative Surgical Procedures OR Procedures, Operative Surgical OR Surgical Procedure, Operative OR Operative Procedures OR Operative Procedure OR Procedure, Operative OR Procedures, Operative OR Procedure, Operative Surgical OR Pancreatectomy OR Pancreatectomies OR Pancreaticoduodenectomy OR Pancreatoduodenectomies OR Duodenopancreatectomy OR Duodenopancreatectomies OR Enucleation	11199
4#	1# AND 2# AND 3#	48

Supplementary Table S2 Quality assessment of cohort studies by NOS

Author	Selection	Comparability	Outcome	Total score
Lee	4	0	3	7
Gratian	3	0	2	5
Jung	4	0	2	6
Regenet	4	0	2	6
Sadot	3	1	2	7
Rosenberg	4	0	2	6
